# Benzene-1,2-dicarb­oxy­lic acid–pyridinium-2-olate (1/1)

**DOI:** 10.1107/S1600536812023914

**Published:** 2012-06-02

**Authors:** Chua-Hua Yu

**Affiliations:** a Orderd Matter Science Research Center, Southeast University, Nanjing 211189, People’s Republic of China

## Abstract

The asymmetric unit of the title compound, C_5_H_5_NO·C_8_H_6_O_4_, contains one *o*-phthalate acid mol­ecule and one pyridin-2-ol mol­ecule, which exists in a zwitterionic form. In the *o*-phthalate acid mol­ecule, the carboxyl­ate groups are twisted from the benzene ring by dihedral angles of 13.6 (1)° and 73.1 (1)°; the hy­droxy H atom in the latter group is disordered over two positons in a 1:1 ratio. In the crystal, O—H⋯O and N—H⋯O hydrogen bonds link the mol­ecules into zigzag chains in [-101].

## Related literature

For background to molecular ferroelectrics, see: Zhang *et al.* (2009 ▶, 2010 ▶, 2012 ▶). For a related structure, see: Zhu & Yu (2011 ▶). 
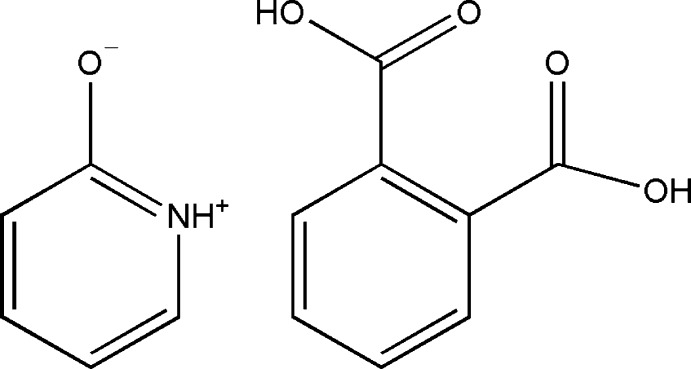


## Experimental

### 

#### Crystal data

C_5_H_5_NO·C_8_H_6_O_4_
*M* *_r_* = 261.23
Triclinic, 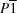

*a* = 7.4529 (15) Å
*b* = 7.7925 (16) Å
*c* = 11.489 (2) Å
α = 84.42 (3)°
β = 84.29 (3)°
γ = 70.30 (3)°
*V* = 623.6 (2) Å^3^
*Z* = 2
Mo *K*α radiation
μ = 0.11 mm^−1^
*T* = 293 K
0.34 × 0.30 × 0.28 mm

#### Data collection

Rigaku SCXmini diffractometer
Absorption correction: multi-scan (*CrystalClear*; Rigaku, 2005 ▶) *T*_min_ = 0.964, *T*_max_ = 0.970
6555 measured reflections
2864 independent reflections
1687 reflections with *I* > 2σ(*I*)
*R*_int_ = 0.034

#### Refinement

*R*[*F* ^2^ > 2σ(*F* ^2^)] = 0.054
*wR*(*F* ^2^) = 0.174
*S* = 1.06
2864 reflections
184 parameters
6 restraints
H atoms treated by a mixture of independent and constrained refinement
Δρ_max_ = 0.20 e Å^−3^
Δρ_min_ = −0.21 e Å^−3^

### 

Data collection: *CrystalClear* (Rigaku, 2005 ▶); cell refinement: *CrystalClear*; data reduction: *CrystalClear*; program(s) used to solve structure: *SHELXS97* (Sheldrick, 2008 ▶); program(s) used to refine structure: *SHELXL97* (Sheldrick, 2008 ▶); molecular graphics: *DIAMOND* (Brandenburg & Putz, 2005 ▶); software used to prepare material for publication: *SHELXL97*.

## Supplementary Material

Crystal structure: contains datablock(s) I, global. DOI: 10.1107/S1600536812023914/cv5304sup1.cif

Structure factors: contains datablock(s) I. DOI: 10.1107/S1600536812023914/cv5304Isup2.hkl

Supplementary material file. DOI: 10.1107/S1600536812023914/cv5304Isup3.cml

Additional supplementary materials:  crystallographic information; 3D view; checkCIF report

## Figures and Tables

**Table 1:** Hydrogen-bond geometry (Å, °)

*D*—H⋯*A*	*D*—H	H⋯*A*	*D*⋯*A*	*D*—H⋯*A*
O3—H3*A*⋯O4^i^	0.86 (2)	1.80 (2)	2.644 (3)	167 (7)
O4—H3*A*′⋯O3^i^	0.85 (3)	1.81 (3)	2.644 (3)	167 (4)
O1—H1*B*⋯O5^ii^	0.85 (2)	1.74 (2)	2.587 (2)	178 (3)
N1—H1*A*⋯O5^iii^	0.86	2.04	2.892 (3)	171

Symmetry codes: (i) 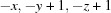; (ii) 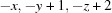; (iii) 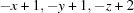.

**Fig. 1.**
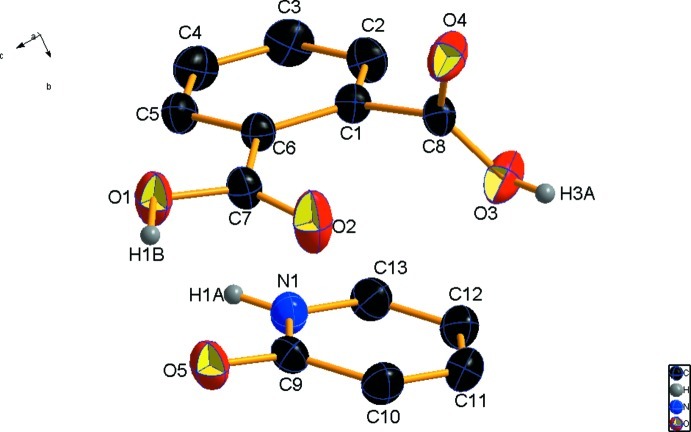
A content of asymmetric unit, with displacement ellipsoids drawn at the 30% probability level. For the disordered atom H3A (attached either to O3 or to O4), only one position is shown. C-bound H atoms omitted for clarity.

**Fig. 2.**
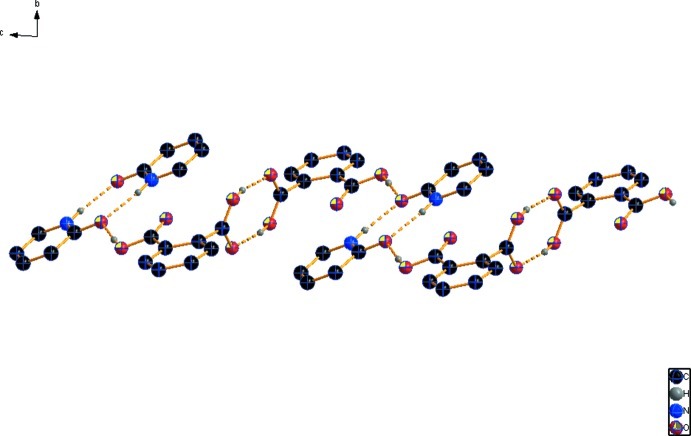
A portion of the crystal packing showing hydrogen-bonded (dashed lines) chain of the molecules. For the disordered hydroxy H atom only one position is shown. C-bound H atoms omitted for clarity.

## supplementary crystallographic information

### Comment

The title compound was synthesized to find potential ferroelectric phase change materials *via* dielectric constant measurements of compounds on the basis of temperature (Zhang, Chen *et al.*, 2009; Zhang, Ye *et al.*, 2010; Zhang & Xiong, 2012), with reference to the compound C_5_H_9_N_2_^+^.C_8_H_5_O_4_^-^ (Zhu & Yu, 2011). Regrettably, no dielectric anomaly was observed ranging from 120 K to 353 K near its melting point. Herewith we report the crystal structure of the title compound, (I).

The asymmetric unit of (I) contains one molecule of the *o*-phthalate acid and one pyridin-2-ol molecule, which exists in a zwitterionic form (Fig. 1). In the *o*-phthalate acid molecule, atom H3A is disordered over two positions being attached either to O3 or to O4 in a ratio 1:1. Intermolecular N—H···O and O—H···O hydrogen bonds (Table 1) link the molecules into zigzag chains in [-1 0 1] (Fig. 2).

### Experimental

0.83 g (5 mmol) of phthalic acid and 10 ml water which were heated, then added with a few ethanol dropst, and 0.476 g (5 mmol) 2-hydroxypyridine was added to the solution. After stirring the mixture for minutes for the sake of achieving the ambient temperature, the liquid was filtered to give a clear solution. Colourless block crystals suitable for X-ray structure analysis were obtained, by the slow evaporation of the above solution after sever days at the ambient temperature.

### Refinement

O-bound H atoms were located on a difference map and isotropically refined with restraint O—H = 0.85 (2) Å. The rest H atoms were placed in geometrically idealized positions (N—H = 0.86 Å; C—H = 0.93 Å) and refined as riding, with *U_iso_*(H) = 1.2 *U_iso_*(C, N).

### Crystal data

**Table:**

C_5_H_5_NO·C_8_H_6_O_4_	*Z* = 2
*M**_r_* = 261.23	*F*(000) = 272
Triclinic, *P*1	*D*_x_ = 1.391 Mg m^−^^3^
Hall symbol: -P 1	Mo *K*α radiation, λ = 0.71073 Å
*a* = 7.4529 (15) Å	Cell parameters from 2864 reflections
*b* = 7.7925 (16) Å	θ = 3.2–27.5°
*c* = 11.489 (2) Å	µ = 0.11 mm^−^^1^
α = 84.42 (3)°	*T* = 293 K
β = 84.29 (3)°	Block, colourless
γ = 70.30 (3)°	0.34 × 0.30 × 0.28 mm
*V* = 623.6 (2) Å^3^	

### Data collection

**Table:**

Rigaku, SCXmini diffractometer	1687 reflections with *I* > 2σ(*I*)
Radiation source: fine-focus sealed tube	*R*_int_ = 0.034
Graphite monochromator	θ_max_ = 27.5°, θ_min_ = 3.2°
ω scans	*h* = −9→9
Absorption correction: multi-scan *CrystalClear* (Rigaku, 2005)	*k* = −10→10
*T*_min_ = 0.964, *T*_max_ = 0.970	*l* = −14→14
6555 measured reflections	3 standard reflections every 180 reflections
2864 independent reflections	intensity decay: none

### Refinement

**Table:**

Refinement on *F*^2^	Primary atom site location: structure-invariant direct methods
Least-squares matrix: full	Secondary atom site location: difference Fourier map
*R*[*F*^2^ > 2σ(*F*^2^)] = 0.054	Hydrogen site location: inferred from neighbouring sites
*wR*(*F*^2^) = 0.174	H atoms treated by a mixture of independent and constrained refinement
*S* = 1.06	*w* = 1/[σ^2^(*F*_o_^2^) + (0.0891*P*)^2^] where *P* = (*F*_o_^2^ + 2*F*_c_^2^)/3
2864 reflections	(Δ/σ)_max_ < 0.001
184 parameters	Δρ_max_ = 0.20 e Å^−^^3^
6 restraints	Δρ_min_ = −0.21 e Å^−^^3^

### Special details

**Table:**

Geometry. All e.s.d.'s (except the e.s.d. in the dihedral angle between two l.s. planes) are estimated using the full covariance matrix. The cell e.s.d.'s are taken into account individually in the estimation of e.s.d.'s in distances, angles and torsion angles; correlations between e.s.d.'s in cell parameters are only used when they are defined by crystal symmetry. An approximate (isotropic) treatment of cell e.s.d.'s is used for estimating e.s.d.'s involving l.s. planes.
Refinement. Refinement of *F*^2^ against ALL reflections. The weighted *R*-factor *wR* and goodness of fit *S* are based on *F*^2^, conventional *R*-factors *R* are based on *F*, with *F* set to zero for negative *F*^2^. The threshold expression of *F*^2^ > σ(*F*^2^) is used only for calculating *R*-factors(gt) *etc*. and is not relevant to the choice of reflections for refinement. *R*-factors based on *F*^2^ are statistically about twice as large as those based on *F*, and *R*- factors based on ALL data will be even larger.

### Fractional atomic coordinates and isotropic or equivalent isotropic displacement parameters (Å^2^)

**Table:**

	*x*	*y*	*z*	*U*_iso_*/*U*_eq_	Occ. (<1)
C1	0.3559 (3)	0.3156 (3)	0.68465 (17)	0.0443 (5)	
C2	0.5446 (3)	0.2808 (3)	0.6408 (2)	0.0567 (6)	
H2	0.5713	0.3150	0.5629	0.068*	
C3	0.6907 (3)	0.1969 (3)	0.7113 (2)	0.0662 (7)	
H3	0.8165	0.1738	0.6811	0.079*	
C4	0.6532 (3)	0.1465 (3)	0.8262 (2)	0.0633 (6)	
H4	0.7537	0.0872	0.8732	0.076*	
C5	0.4692 (3)	0.1827 (3)	0.87225 (18)	0.0532 (6)	
H5	0.4452	0.1500	0.9508	0.064*	
C6	0.3168 (3)	0.2685 (3)	0.80241 (17)	0.0433 (5)	
C7	0.1172 (3)	0.3228 (3)	0.85256 (18)	0.0508 (5)	
C8	0.2024 (3)	0.3968 (3)	0.60303 (16)	0.0456 (5)	
C9	0.3260 (3)	0.6814 (3)	0.87731 (18)	0.0491 (5)	
C10	0.2091 (3)	0.8029 (3)	0.7956 (2)	0.0623 (6)	
H10	0.0770	0.8389	0.8093	0.075*	
C11	0.2866 (4)	0.8680 (3)	0.6973 (2)	0.0665 (7)	
H11	0.2069	0.9480	0.6440	0.080*	
C12	0.4816 (4)	0.8177 (3)	0.6746 (2)	0.0632 (6)	
H12	0.5342	0.8633	0.6068	0.076*	
C13	0.5943 (3)	0.7015 (3)	0.7522 (2)	0.0611 (6)	
H13	0.7264	0.6659	0.7385	0.073*	
H3A	0.063 (5)	0.612 (8)	0.532 (5)	0.11 (2)*	0.50
H3A'	0.038 (5)	0.352 (5)	0.521 (3)	0.11 (2)*	0.50
H1B	−0.026 (3)	0.294 (4)	0.983 (2)	0.092 (10)*	
N1	0.5161 (3)	0.6359 (2)	0.85064 (15)	0.0547 (5)	
H1A	0.5917	0.5613	0.8987	0.066*	
O1	0.0910 (3)	0.2509 (2)	0.95711 (14)	0.0729 (5)	
O2	−0.0108 (2)	0.4285 (3)	0.79990 (16)	0.0876 (6)	
O3	0.1680 (3)	0.5598 (2)	0.56473 (16)	0.0723 (5)	
O4	0.1233 (3)	0.2930 (2)	0.56793 (15)	0.0696 (5)	
O5	0.2656 (2)	0.6139 (2)	0.97148 (13)	0.0663 (5)	

### Atomic displacement parameters (Å^2^)

**Table:**

	*U*^11^	*U*^22^	*U*^33^	*U*^12^	*U*^13^	*U*^23^
C1	0.0418 (11)	0.0481 (11)	0.0449 (11)	−0.0178 (9)	−0.0053 (9)	0.0001 (9)
C2	0.0510 (13)	0.0692 (15)	0.0519 (12)	−0.0261 (11)	0.0056 (11)	−0.0011 (11)
C3	0.0394 (12)	0.0793 (17)	0.0794 (17)	−0.0192 (12)	−0.0011 (12)	−0.0083 (13)
C4	0.0457 (14)	0.0684 (15)	0.0716 (16)	−0.0089 (11)	−0.0185 (12)	−0.0064 (12)
C5	0.0539 (13)	0.0566 (13)	0.0462 (12)	−0.0129 (10)	−0.0119 (10)	−0.0016 (10)
C6	0.0418 (11)	0.0464 (11)	0.0414 (11)	−0.0142 (9)	−0.0052 (9)	0.0000 (8)
C7	0.0486 (13)	0.0613 (13)	0.0412 (11)	−0.0177 (11)	−0.0029 (10)	0.0010 (10)
C8	0.0490 (12)	0.0524 (13)	0.0377 (10)	−0.0221 (10)	−0.0026 (9)	0.0045 (9)
C9	0.0498 (12)	0.0537 (12)	0.0409 (11)	−0.0147 (10)	0.0057 (9)	−0.0065 (9)
C10	0.0552 (14)	0.0587 (14)	0.0626 (14)	−0.0072 (11)	−0.0008 (11)	−0.0015 (11)
C11	0.0803 (18)	0.0523 (14)	0.0582 (15)	−0.0121 (12)	−0.0107 (13)	0.0073 (11)
C12	0.0769 (17)	0.0550 (13)	0.0524 (13)	−0.0210 (12)	0.0078 (12)	0.0058 (11)
C13	0.0585 (14)	0.0608 (14)	0.0580 (14)	−0.0169 (11)	0.0108 (11)	0.0000 (11)
N1	0.0523 (11)	0.0583 (11)	0.0472 (10)	−0.0121 (9)	−0.0012 (9)	0.0028 (8)
O1	0.0565 (11)	0.0911 (13)	0.0538 (10)	−0.0116 (9)	0.0091 (8)	0.0177 (9)
O2	0.0456 (10)	0.1286 (16)	0.0627 (11)	−0.0037 (10)	−0.0012 (8)	0.0241 (10)
O3	0.0820 (13)	0.0609 (11)	0.0843 (12)	−0.0359 (9)	−0.0379 (10)	0.0231 (9)
O4	0.0824 (12)	0.0601 (10)	0.0769 (12)	−0.0331 (9)	−0.0393 (10)	0.0142 (9)
O5	0.0584 (10)	0.0838 (11)	0.0494 (9)	−0.0192 (8)	0.0075 (8)	0.0033 (8)

### Geometric parameters (Å, º)

**Table:**

C1—C2	1.391 (3)	C9—O5	1.266 (2)
C1—C6	1.396 (3)	C9—N1	1.351 (3)
C1—C8	1.483 (3)	C9—C10	1.404 (3)
C2—C3	1.365 (3)	C10—C11	1.350 (3)
C2—H2	0.9300	C10—H10	0.9300
C3—C4	1.370 (4)	C11—C12	1.377 (3)
C3—H3	0.9300	C11—H11	0.9300
C4—C5	1.365 (3)	C12—C13	1.342 (3)
C4—H4	0.9300	C12—H12	0.9300
C5—C6	1.395 (3)	C13—N1	1.355 (3)
C5—H5	0.9300	C13—H13	0.9300
C6—C7	1.475 (3)	N1—H1A	0.8600
C7—O2	1.201 (3)	O1—H1B	0.85 (2)
C7—O1	1.300 (2)	O3—H3A	0.855 (15)
C8—O3	1.252 (2)	O4—H3A'	0.85 (3)
C8—O4	1.263 (2)		
			
C2—C1—C6	119.45 (19)	O4—C8—C1	117.90 (18)
C2—C1—C8	118.47 (18)	O5—C9—N1	119.3 (2)
C6—C1—C8	122.05 (17)	O5—C9—C10	124.9 (2)
C3—C2—C1	120.4 (2)	N1—C9—C10	115.89 (19)
C3—C2—H2	119.8	C11—C10—C9	120.7 (2)
C1—C2—H2	119.8	C11—C10—H10	119.7
C2—C3—C4	120.4 (2)	C9—C10—H10	119.7
C2—C3—H3	119.8	C10—C11—C12	121.1 (2)
C4—C3—H3	119.8	C10—C11—H11	119.5
C5—C4—C3	120.4 (2)	C12—C11—H11	119.5
C5—C4—H4	119.8	C13—C12—C11	118.6 (2)
C3—C4—H4	119.8	C13—C12—H12	120.7
C4—C5—C6	120.5 (2)	C11—C12—H12	120.7
C4—C5—H5	119.7	C12—C13—N1	120.2 (2)
C6—C5—H5	119.7	C12—C13—H13	119.9
C5—C6—C1	118.79 (19)	N1—C13—H13	119.9
C5—C6—C7	121.36 (18)	C9—N1—C13	123.6 (2)
C1—C6—C7	119.67 (18)	C9—N1—H1A	118.2
O2—C7—O1	122.9 (2)	C13—N1—H1A	118.2
O2—C7—C6	121.49 (18)	C7—O1—H1B	110 (2)
O1—C7—C6	115.58 (19)	C8—O3—H3A	117 (4)
O3—C8—O4	122.96 (19)	C8—O4—H3A'	111 (3)
O3—C8—C1	118.87 (18)		

### Hydrogen-bond geometry (Å, º)

**Table:**

*D*—H···*A*	*D*—H	H···*A*	*D*···*A*	*D*—H···*A*
O3—H3*A*···O4^i^	0.86 (2)	1.80 (2)	2.644 (3)	167 (7)
O4—H3*A*′···O3^i^	0.85 (3)	1.81 (3)	2.644 (3)	167 (4)
O1—H1*B*···O5^ii^	0.85 (2)	1.74 (2)	2.587 (2)	178 (3)
N1—H1*A*···O5^iii^	0.86	2.04	2.892 (3)	171

Symmetry codes: (i) −*x*, −*y*+1, −*z*+1; (ii) −*x*, −*y*+1, −*z*+2; (iii) −*x*+1, −*y*+1, −*z*+2.
